# A retrospective clinical comparison of daptomycin vs daptomycin and a beta-lactam antibiotic for treating vancomycin-resistant *Enterococcus faecium* bloodstream infections

**DOI:** 10.1038/s41598-018-19986-8

**Published:** 2018-01-26

**Authors:** Yu-Chung Chuang, Pao-Yu Chen, Chi-Ying Lin, Yee-Chun Chen, Jann-Tay Wang, Shan-Chwen Chang

**Affiliations:** 1 Graduate Institute of Clinical Medicine, College of Medicine, National Taiwan University, Taipei, Taiwan; 2 Department of Internal Medicine, National Taiwan University Hospital, Taipei, Taiwan; 3 Department of Traumatology, National Taiwan University Hospital, Taipei, Taiwan; 4 Department of Internal Medicine, National Taiwan University Hospital Yun-Lin Branch, Yun-Lin, Taiwan

## Abstract

There is limited clinical evidence to support the combination of daptomycin and beta-lactam antibiotics (DAP + BLA) for treatment of vancomycin-resistant enterococci (VRE) bloodstream infections (BSI). We conducted a prospective observational cohort study of VRE-BSI during 2010–2015. The primary endpoint was mortality at the end of treatment. We included 114 patients who received DAP for VRE-BSI. Of these 87 (76.3%) received DAP + BLA. There were no significant differences in mortality between the DAP and DAP + BLA groups on univariable analysis (10/27 *vs*. 34/87, *P* = 0.85). A subgroup analysis of patients with enterococcal DAP minimum inhibitory concentrations (MICs) ≤2 mg/L, revealed that those treated with DAP + BLA had a lower mortality (adjusted hazard ratio [aHR], 0.23; 95% confidence interval [CI], 0.06–0.93; *P* = 0.04) after adjustment for other significant predictors of mortality, including the DAP dose. In addition, patients receiving high-dose (≥9 mg/kg) DAP + BLA independently had a better survival than those receiving low-dose DAP alone (aHR = 5.16), low-dose DAP + BLA (aHR = 5.39), and high-dose DAP alone (aHR = 19.01) (*P* < 0.05 for all comparisons). For patients with VRE-BSIs, the DAP MIC of the isolate and the DAP dose influence the effect of DAP + BLA on outcome. A high-dose DAP + BLA might improve survival. These findings support the use of high-dose DAP + BLA for treatment of VRE-BSI.

## Introduction

Vancomycin resistant enterococci (VRE) are important causes of healthcare-associated infections^1^. Antimicrobial treatment options are limited^2^. Although the US Food and Drug Administration has not yet approved daptomycin (DAP) for treatment of VRE infections, clinicians are increasingly using DAP for VRE bloodstream infections (VRE-BSIs)^3,4^. While higher DAP doses improve the therapeutic response^5,6^, the mortality rate from VRE-BSI remains high^5,7^.

Beta-lactam antibiotics (BLAs) reduce the net positive bacterial surface charge of VRE, and thereby enhance the bactericidal effect of DAP^8–10^. Several studies have found an *in vitro* synergistic effect between DAP and various BLAs^8–13^. Case reports have shown that combinations of DAP and a BLA (DAP + BLA) can cure infective endocarditis with persistent enterococcal BSI^14,15^. Based on these results, some investigators recommend that DAP + BLA be used for the treatment of VRE infections^16,17^. Unfortunately the few cohort studies that have evaluated the clinical or microbiological effects of DAP + BLA for treatment of VRE-BSI have been inconclusive^5,18,19^. This may be due to a number of confounders. These include the dose of DAP and the minimum inhibitory concentration (MIC) of VRE isolates. Both of these are associated with clinical^5,6^ and microbiological outcomes^18^. These factors might also confound the synergistic effect of DAP + BLA^9,10^. Namely, the DAP doses and DAP MICs were not evaluated when DAP + BLA cohort studies were reported^5,18,19^.

The primary aim of the current observational study in patients with VRE-BSIs was to determine whether DAP + BLA were more effective than DAP alone on patient survival. The secondary aim was to determine the influence of the DAP MIC and DAP dose on the effect of DAP + BLA compared to DAP alone.

## Patients and Methods

### Hospital Setting and Patient Selection

The study was conducted in accordance with the principles expressed in the Declaration of Helsinki at the National Taiwan University Hospital (NTUH), a 2200-bed medical center located in Taipei City, and NTUH Yun-Lin Branch, a 600-bed regional teaching hospital in Yun-Lin County. Patients with VRE-BSIs were enrolled from January 2010 through July 2015. The analysis was performed after approval by the Research Ethics Committee of the NTUH (NTUH 201606064RINB). The Board waived the need for informed consent, since this study required no additional intervention and all the data were analyzed anonymously. In order to determine whether DAP + BLA was more effective than DAP alone on patient survival, the analysis of data in the current study are different from a previous study of the same population^7^, and there is little overlap. In the present study, we used mortality at the end of daptomycin treatment as the primary end-point. We used Cox or time-dependent Cox regression for outcome analysis to handle the immortal-time bias. We evaluated the effect of the interaction between daptomycin dose and the BLA combinations on survival. On the other hand, in the previous study, we aimed to determine whether higher doses of daptomycin were more effective than customary doses. In that study, we used 14-day mortality as the primary end-point and evaluated using logistic regression. However, the effect of the interaction between daptomycin dose and the BLA combinations on survival was not investigated in the previous study.

A VRE-BSI was defined as growth of VRE in blood culture. If a patient had multiple VRE-BSI episodes during the study period, only the first episode was included. All included patients received a DAP dose of at least 6 mg/kg. The included patients received DAP as the first VRE-BSI treatment and subsequently completed the treatment course with DAP. Patients were excluded if they were younger than 18 years-old, not admitted to the hospital, had no VRE isolates available for DAP MIC testing, received another *in vitro* active antimicrobial agent (other than an aminoglycoside) against the causative VRE isolate, or had polymicrobial BSI and received inappropriate antimicrobial therapy as determined by *in vitro* susceptibility results for the non-VRE pathogen(s).

### Microbiological Studies and Antimicrobial Susceptibility Testing

Blood cultures were processed by the clinical microbiology laboratory. VRE was identified using the VITEK 2 identification system (bioMérieux Inc., La Balme les Grottes, France). Vancomycin resistance was defined as a MIC of at least 32 mg/L. VRE isolates collected at the two hospitals were stored at −80 °C until use. MIC tests of all available isolates were performed at the NTUH. The DAP MICs of enterococci were determined using the broth microdilution method and interpreted according to the Clinical and Laboratory Standards Institute^20^. Cation-adjusted Mueller-Hinton broth (Becton Dickinson, Le Pont-de-Claix, France), with 50 μg/mL of supplemented calcium, was used for these tests.

### Clinical Data Collection and Definitions

We prospectively recorded demographic data, underlying diseases, sites of infection^21^, and all causes of in-hospital mortality. Catheter-related BSIs and central line-associated BSIs (CLABSIs) were defined according to previous definitions^22^. The Charlson comorbidity index (CCI) was used to adjust for underlying conditions^23^. BSI severity was assessed using the Pitt bacteremia score (PBS) at the onset of BSI^24^.

A DAP + BLA regimen was defined as the receipt of at least one dose of a BLA during the course of DAP treatment^5,19^. Patients who received DAP + BLA regimen were classified as the DAP + BLA group, and patients who received DAP without a BLA as the DAP group. The primary care physicians determined the DAP dose and administration of a BLA. Taiwan currently has no local guidelines regarding the DAP dose or combined use of a BLA for treatment of VRE-BSI. The reasons of combined use of a BLA might be empirical treatment for sepsis, or treatment for other infections such as pneumonia or urinary tract infection. A DAP dose of at least 9 mg/kg was defined as “high-dose”, and a dose of 6–9 mg/kg as “low-dose”^6^.

The date of BSI onset was defined as the day when the blood samples of VRE-positive blood culture was drawn. Use of immunosuppressive agents was defined as receipt of antineoplastic drugs, cyclophosphamide, or other immunosuppressive agents within 6 weeks, or receipt of prednisolone at a dosage of 20 mg/day for 2 or more weeks or 30 mg/day for 1 or more weeks before BSI onset. Thrombocytopenia was defined as a platelet count below 50,000/μL^19^. The NTUH recommends that creatine phosphokinase (CPK) level be measured at least once per week during DAP treatment^25^ or if symptoms appear. An elevated CPK level was defined as above the normal upper limit^26^. The primary outcome was mortality at the end of DAP treatment. The secondary outcomes were overall in-hospital mortality and elevated CPK level.

### Statistical Analysis

Medians and the interquartile ranges (IQRs) were calculated for continuous variables, and percentages were calculated for categorical variables. Continuous variables were compared using a Mann–Whitney *U* test and categorical variables using a χ^2^ test or a 2-tailed Fisher’s exact test, as appropriate. Kaplan-Meier curves were used for survival analysis. Multivariable Cox proportional hazard analyses were performed to analyze outcomes. A variable with a *P* value of 0.1 or less in the univariable analysis was included in the multivariable analysis. Multivariable models were developed using a backward, stepwise method, with minimization of the Akaike information criterion (AIC)^27^. Following the stepwise AIC selection, only variables with *P* values of 0.05 or less were considered significant and included in the final model. The effect of DAP MIC and DAP dose on DAP + BLA treatment were also evaluated by stratified analysis, and consideration of the interaction term in the final model. Sensitivity analysis was conducted using time-dependent analysis to avoid the possible “immortal time” bias of DAP + BLA^28^. DAP or DAP + BLA was treated as a right-censored time-dependent variable in the time-dependent multivariable Cox proportional hazard analysis. Stata software (v. 14; StataCorp, College Station, TX) was used for statistical analyses and a 2-sided *P* values of 0.05 or less were considered significant.

### Data Availability

The datasets generated during and/or analysed during the current study are available from the corresponding author on reasonable request.

## Results

### Patient selection, baseline characteristics, and comparison of patient groups

**Figure 1:**
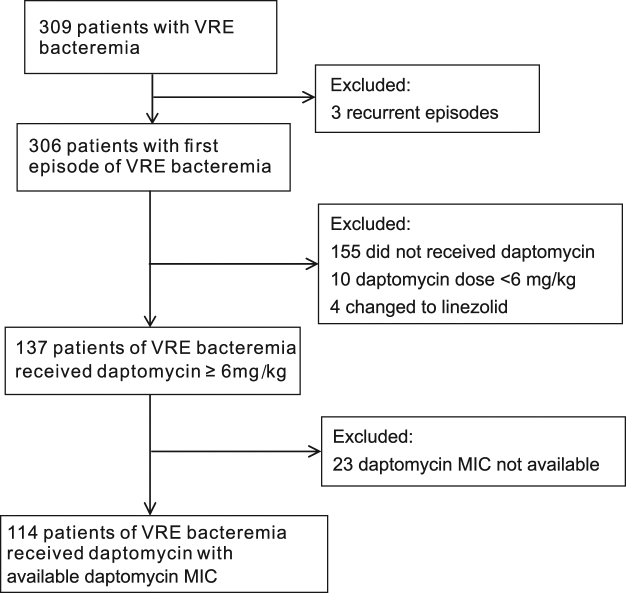
Identification and selection of the 114 patients with VRE-BSI who received DAP with or without a BLA and had DAP MIC data available.

During the study period, we identified 309 episodes of VRE-BSI. One hundred and fourteen patients met the inclusion criteria and had DAP MIC data available (Fig. 1). All VRE isolates were *E. faecium*, and all were resistant to ampicillin. One hundred and three (91.2%) VRE isolates had an ampicillin MIC of ≥256 mg/L. Among the VRE isolates, 2 (1.8%) were considered resistant to DAP, with a DAP MICs of 8 mg/L. There were 78 isolates (68.4%) with MICs of 4 mg/L, 29 (25.4%) with MICs of 2 mg/L, and 5 (4.4%) with MICs of 1 mg/L.

The median age of the study cohort was 65.4 years (IQR: 53.5–78.2) (Table 1). Sixty-one patients (53.5%) were men. The median DAP dose was 7.8 mg/kg (IQR: 6.8–8.7). Four patients had at least one dose of a concomitant aminoglycoside. There were 87 patients in the DAP + BLA group; 10 (8.8%) received a penicillin, 41 (36.0%) received a cephalosporin, and 62 (54.4%) received a carbapenem. The median duration of BLA combination was 9 days (IQR: 5–13). We summarized a table which described the BLA therapy for each of the 87 patients (Supplementary Table S1). The DAP + BLA group didn’t have a significantly lower ampicillin MIC distribution (*P* = 0.86). None of the combination group received the cefazolin or cefotaxime as the combined BLAs. Forty-four (38.6%) patients died at the end of DAP treatment. The overall in-hospital mortality rate was 72/114 (63.2%).

**Table 1:** Demographic and Clinical Characteristics of Patients with Vancomycin-Resistant Enterococcal Bloodstream Infection.

Variable^a^	Total (n = 114)	DAP (n = 27)	DAP + BLA (n = 87)	*P* ^a^
**Demographics**				
Age (years)	65.4 (53.5–78.2)	62.6 (49.8–75.1)	67.8 (54.8–80.5)	0.23
Male	61 (53.5)	18 (66.7)	43 (49.4)	0.12
Body weight (kg)	58.1 (50.8–64.9)	61.5 (52.6–66.5)	57.8 (49.9–63.8)	0.10
Duration of prior hospitalization (days)	24 (10–42)	24 (17–66)	24 (9–38)	0.11
**Underlying conditions**				
Charlson comorbidity score	3 (2–5)	3 (2–6)	3 (2–5)	0.82
Autoimmune disease	4 (3.5)	0 (0)	4 (4.6)	0.57
Liver cirrhosis	18 (15.8)	3 (11.1)	15 (17.2)	0.56
Diabetes mellitus	32 (28.1)	4 (14.8)	28 (32.1)	0.08
Chronic kidney disease	35 (30.7)	6 (22.2)	29 (33.3)	0.27
Any Malignancy	62 (54.4)	12 (44.4)	50 (57.5)	0.24
Solid tumor	31 (27.4)	4 (15.4)	27 (31.0)	0.12
Hematologic	30 (26.3)	10 (37.0)	20 (23.0)	0.15
Metastatic	15 (13.2)	6 (22.2)	9 (10.3)	0.19
Use of immunosuppressive agents	54 (47.4)	17 (63.0)	37 (42.5)	0.06
Steroid	18 (15.8)	6 (22.2)	12 (13.8)	0.37
Chemotherapy	39 (34.2)	14 (51.9)	25 (28.7)	0.03
Solid organ transplant recipient	2 (1.8)	0 (0)	2 (2.3)	0.99
Hematopoietic stem cell transplant recipient	7 (6.1)	2 (7.4)	5 (5.7)	0.67
**Infection focus**				
CLABSI	50 (43.9)	14 (51.9)	36 (41.4)	0.34
CRBSI	7 (6.1)	1 (3.7)	6 (6.9)	0.99
Urinary tract infection	20 (17.5)	5 (18.5)	15 (17.2)	0.99
Intra-abdominal infection	11 (9.6)	1 (3.7)	10 (11.5)	0.46
Surgical site infection	5 (4.4)	1 (3.7)	4 (4.6)	0.99
Unknown	25 (21.9)	5 (18.5)	20 (23.0)	0.62
**Clinical characteristics**				
Neutropenia (ANC < 500/μL)	30 (26.3)	8 (29.6)	22 (25.3)	0.65
Platelet count (×10^4^/µL)	7.8 (2.9–17)	10.5 (2.6–21)	7.7 (2.9–16.7)	0.87
Thrombocytopenia (<50000/μL)	35 (30.7)	9 (33.3)	26 (29.9)	0.73
Ventilator use	47 (41.2)	12 (44.4)	35 (40.2)	0.70
Pitt bacteremia score	3 (1–6)	3 (1–6)	3 (1–6)	0.92
Concomitant aminoglycoside use	4 (3.5)	2 (7.4)	2 (2.3)	0.24
DAP dose (mg/kg)	7.8 (6.8–8.7)	7.7 (6.3–8.3)	7.8 (6.8–9.1)	0.33
DAP dose ≥9 mg/kg	26 (22.8)	4 (14.8)	22 (25.3)	0.26
DAP MIC 1 mg/L	5 (4.4)	0 (0)	5 (5.8)	0.69
DAP MIC 2 mg/L	29 (25.4)	7 (25.9)	22 (25.3)	
DAP MIC ≥4 mg/L	80 (70.2)	20 (74.1)	60 (69.0)	
**Outcomes**				
Mortality at the end of daptomycin treatment	44 (38.6)	10 (37.4)	34 (39.1)	0.85
In-hospital mortality	72 (63.2)	16 (59.3)	56 (64.4)	0.63
Elevated creatinine kinase	7 (6.1)	1 (3.7)	6 (6.9)	0.99

Abbreviations: ANC, absolute neutrophil count; BLA, beta-lactam antibiotic; CLABSI, central line-associated bloodstream infection; CRBSI, catheter-related bloodstream infection; DAP, daptomycin; MIC, minimum inhibitory concentration.

^a^Data are presented as median (IQR) for continuous variables and N (%) for categorical variables. The Mann-Whitney U test was used to compare continuous variables, and the χ^2^ or 2-tailed Fisher’s exact test to compare categorical variables.

The DAP and the DAP + BLA groups had similar baseline characteristics (Table 1). The only significant difference between the DAP and DAP + BLA groups was that patients in the DAP group had a higher percentage of recent chemotherapy (51.9% *vs*. 28.7%; *P* = 0.03). Notably, the 2 groups had similar PBSs (*P* = 0.92), CCI scores (*P* = 0.82), DAP doses (*P* = 0.33), and DAP MICs (*P* = 0.69) (Table 1). Univariable analysis indicated that the 2 groups also had similar mortality at the end of DAP treatment (*P* = 0.85), overall in-hospital mortality (*P* = 0.63), and elevation of CPK (*P* = 0.99).

We also used univariable analysis to compare survivors and non-survivors (Table 2). The survivors had a higher platelet count (9.4 × 10^4^/µL *vs*. 5.9 × 10^4^/µL; *P* = 0.006), lower prevalence of thrombocytopenia (20.0% *vs*. 47.7%; *P* = 0.002), lower use of steroids (7.1% *vs*. 29.5%; *P* = 0.001), lesser disease severity as indicated by PBS (2 *vs*. 4; *P* = 0.008), and received a higher DAP dose (7.9 *vs*. 7.1 mg/kg; *P* = 0.01). Among the DAP + BLA group, the non-survivors received shorter BLA combinations compared to the survivors (6 days (IQR: 2–9) vs. 12 days (IQR: 8–14), *P* < 0.001). However, the percentage of patients receiving DPA + BLA were similar among survivors and non-survivors (75.7% *vs*. 77.3%; *P* = 0.85).

**Table 2:** Univariable Cox Proportional Hazards Model of Factors Associated with Mortality in Patients with Vancomycin-Resistant Enterococcal Bloodstream Infection.

Variable^a^	Survival (n = 70)	Mortality (n = 44)	*P* ^a^	**Univariable Cox analysis**	
				HR (95% CI)	*P*
**Demographics**					
Age (years)	64.2 (54.7–80.5)	66.5 (50.6–76.6)	0.78	1.00 (0.99–1.02)	0.72
Male	38 (54.3)	23 (52.3)	0.83	1.07 (0.59–1.95)	0.83
Body weight (kg)	57.6 (51–63.4)	59.4 (49.5–66.3)	0.42	1.02 (0.99–1.04)	0.27
Days of prior hospitalization	23 (9–40)	25 (12–44)	0.63	1.00 (0.99–1.01)	0.72
**Underlying condition**					
Charlson comorbidity score	3 (2–5)	4 (2–5)	0.68	1.04 (0.92–1.16)	0.56
Autoimmune disease	2 (2.9)	2 (4.5)	0.64	1.08 (0.26–4.47)	0.92
Liver cirrhosis	12 (17.1)	6 (13.6)	0.62	0.83 (0.35–1.98)	0.68
Diabetes mellitus	20 (28.6)	12 (27.3)	0.88	1.03 (0.53–2.00)	0.93
Chronic kidney disease	20 (28.6)	15 (34.1)	0.53	1.10 (0.59–2.06)	0.76
Any Malignancy	38 (54.3)	24 (54.5)	0.98	0.95 (0.52–1.72)	0.86
Solid tumor	17 (24.6)	14 (31.8)	0.40	1.18 (0.62–2.22)	0.62
Hematologic	17 (24.3)	13 (29.5)	0.54	1.13 (0.59–2.16)	0.71
Metastatic	10 (14.3)	5 (11.4)	0.65	0.91 (0.36–2.31)	0.84
Use of immunosuppressive agents	30 (42.9)	24 (54.5)	0.22	1.42 (0.78–2.57)	0.25
Steroid	5 (7.1)	13 (29.5)	0.001	3.58 (1.85–6.94)	<0.001
Chemotherapy	25 (35.7)	14 (31.8)	0.67	0.85 (0.45–1.61)	0.62
Solid organ transplant recipient	2 (2.9)	0 (0)	0.52	n.a.	
Hematopoietic stem cell transplant recipient	6 (8.6)	1 (2.3)	0.25	0.24 (0.33–1.77)	0.16
**Infection focus**					
CLABSI	30 (42.9)	20 (45.5)	0.79	1.19 (0.66–2.15)	0.57
CRBSI	3 (4.3)	4 (9.1)	0.43	1.54 (0.55–4.32)	0.41
Urinary tract infection	9 (12.9)	11 (25)	0.10	1.70 (0.85–3.37)	0.13
Intra-abdominal infection	8 (11.4)	3 (6.8)	0.53	0.60 (0.19–1.96)	0.40
Surgical site infection	4 (5.7)	1 (2.3)	0.65	0.39 (0.05–2.87)	0.36
Unknown	19 (27.1)	6 (13.6)	0.09	0.50 (0.21–1.18)	0.11
**Clinical characteristic**					
Neutropenia (ANC <500/μL)	20 (28.6)	10 (22.7)	0.49	0.62 (0.30–1.28)	0.19
Platelet count (×10^4^/µL)	9.4 (5.4–21)	5.9 (1.6–13.9)	0.006	0.96 (0.93–1.00)	0.03
Thrombocytopenia (<50000/μL)	14 (20.0)	21 (47.7)	0.002	2.35 (1.30–4.25)	0.005
Ventilator use	26 (37.1)	21 (47.7)	0.26	1.53 (0.85–2.77)	0.16
Pitt bacteremia score	2 (0–4)	4 (2–8)	0.008	1.17 (1.06–1.30)	0.003
DAP dose (mg/kg)	7.9 (7–9.2)	7.1 (6.4–8.2)	0.01	0.78 (0.62–0.98)	0.03
DAP dose ≥9 mg/kg	20 (28.6)	6 (13.6)	0.06	0.48 (0.20–1.14)	0.10
DAP MIC >2 mg/L	50 (71.4)	30 (68.2)	0.71	0.91 (0.48–1.71)	0.77
Concomitant aminoglycoside use	3 (4.3)	1 (2.3)	0.99	0.45 (0.06–3.31)	0.44
BLA	53 (75.7)	34 (77.3)	0.85	0.89 (0.44–1.81)	0.75
Penicillins	5 (7.1)	5 (11.4)	0.51	1.53 (0.60–3.92)	0.37
Cephalosporins	30 (42.9)	11 (25.0)	0.05	0.47 (0.24–0.93)	0.03
Carbapenems	33 (47.1)	29 (65.9)	0.05	1.63 (0.97–3.05)	0.13

Abbreviations: ANC, absolute neutrophil count; BLA, beta-lactam antibiotic; CI, confidence interval; CLABSI, central line-associated bloodstream infection; CRBSI, catheter-related bloodstream infection; DAP, daptomycin; HR, hazard ratio; MIC, minimum inhibitory concentration; n.a., not applicable

^a^Data are presented as median (IQR) for continuous variables and N (%) for categorical variables. The Mann-Whitney U test was used to compare continuous variables, and the χ^2^ or 2-tailed Fisher’s exact test to compare categorical variables.

**Figure 2:**
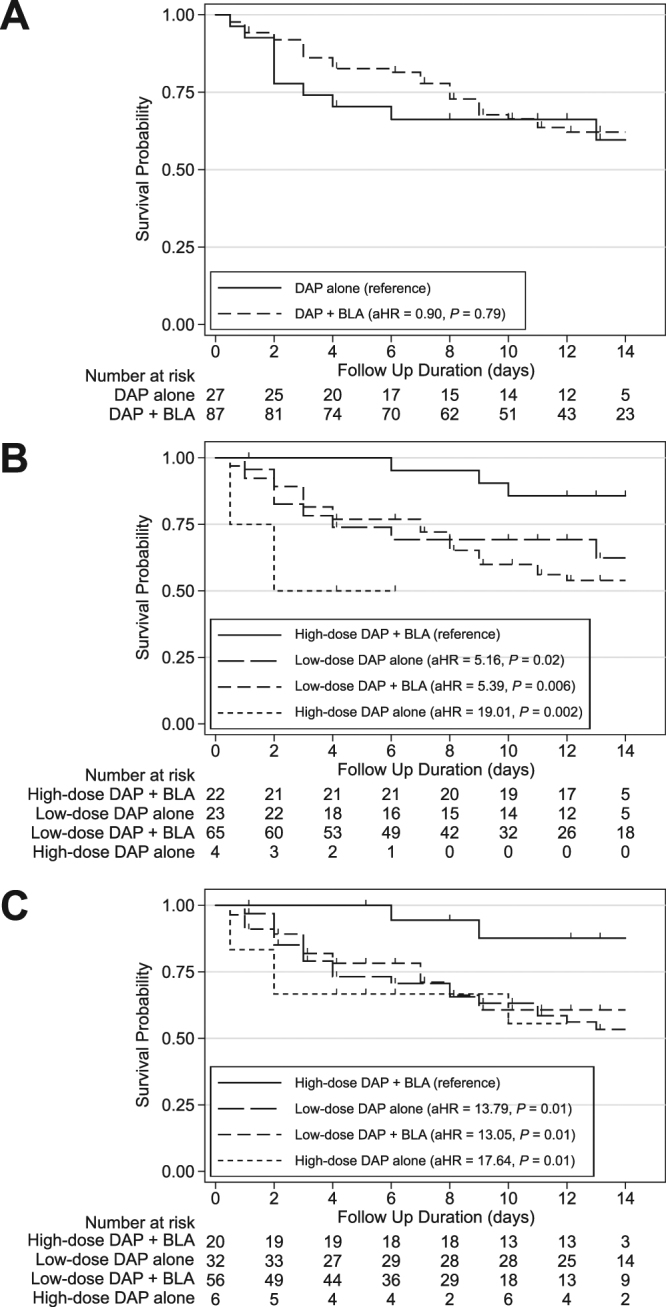
(**A**) Kaplan–Meier survival curves of patients receiving DAP or DAP + BLA. (**B**) Kaplan–Meier survival curves of patients receiving different antibiotic treatments. The group receiving high-dose DAP + BLA had significantly better survival than the other 3 groups (low-dose DAP + BLA, high-dose DAP alone, and low-dose DAP alone). (**C**) Kaplan–Meier survival curves of patients receiving different antibiotic treatments. DAP or DAP + BLA was treated as a right-censored time-dependent variable. The high-dose DAP + BLA group had significantly better survival than the low-dose DAP + BLA group, the high-dose DAP alone group, and the low-dose DAP alone group.

Multivariable Cox proportional hazard model analysis showed that steroid use (adjusted hazard ratio [aHR], 2.86; 95% confidence interval ([CI], 1.42–5.79; *P* = 0.003), PBS (aHR, 1.17; 95% CI, 1.05–1.30; *P* = 0.004), platelet count (aHR, 0.96; 95% CI, 0.92–0.99; *P* = 0.02), and DAP dose (aHR, 0.74; 95% CI, 0.58–0.93; *P* = 0.01) were significant independent predictors of mortality. However, use of DAP + BLA therapy had no significant effect on mortality (aHR, 0.90; 95% CI, 0.41–1.96; *P* = 0.79) (Model 1 in Table 3, and Fig. 2A).

**Table 3:** Multivariable Cox Proportional Hazards Model of Factors Associated with Mortality.

	Adjusted Model 1^a^			Adjusted Model 2^c,d^	
	Hazard ratio (95% CI)	*P*		Hazard ratio (95% CI)	*P*
Steroid use	2.86 (1.42–5.79)	0.003	Steroid use	3.28 (1.64–6.57)	0.001
Pitt bacteremia score	1.17 (1.05–1.30)	0.004	Pitt bacteremia score	1.17 (1.05–1.30)	0.005
Platelet count (×10^4^/μL)	0.96 (0.92–0 0.99)	0.02	Platelet count (×10^4^/μL)	0.96 (0.92–0.99)	0.02
Treatment regimens					
DAP dose (mg/kg)	0.74 (0.58–0.93)	0.01	DAP dose ≥9 mg/kg with BLA	Reference	
DAP + BLA	0.90 (0.41–1.96)	0.79^b^	DAP dose <9 mg/kg without BLA	5.16 (1.34–19.89)	0.02
			DAP dose <9 mg/kg with BLA	5.39 (1.62–17.93)	0.006
			DAP dose ≥9 mg/kg without BLA	19.01 (2.96–121.95)	0.002

Abbreviations: BLA, beta-lactam antibiotic; CI, confidence interval; DAP, daptomycin.

^a^Test of proportional-hazards assumption: *P* = 0.92.

^b^DAP + BLA was forced as an independent variable in the final adjusted model 1.

^c^Test of proportional-hazards assumption: *P* = 0.73.

^d^Interactions between daptomycin dose and beta-lactam combinations were considered in the final adjusted model 2.

### Influence of DAP MIC on the effect of DAP + BLA

There was no significant association between patient mortality and a DAP MIC > 2 mg/L. Therefore, we examined the influence of DAP MIC on the effect of DAP + BLA by stratified the cohort according to the DAP MIC. Among patients whose DAP MICs were 2 mg/L or less, those given DAP + BLA had a significantly lower mortality (aHR, 0.23; 95% CI, 0.06–0.93; *P* = 0.04), even after adjustment for factors such as steroid use (aHR, 5.69; 95% CI, 1.04–31.02; *P* = 0.04), PBS (aHR, 1.42; 95% CI, 1.09–1.85; *P* = 0.009), platelet count (aHR, 0.90; 95% CI, 0.83–0.98; *P* = 0.02), and DAP dose (aHR, 0.72; 95% CI, 0.47–1.11; *P* = 0.14). There was no survival benefit for the DAP + BLA regimen (aHR, 1.46; 95% CI, 0.54–3.97; *P* = 0.47) for patients with DAP MICs of 4 mg/L or more after adjustment for steroid use (aHR, 3.07; 95% CI, 1.34–7.02; *P* = 0.008), PBS (aHR, 1.15; 95% CI, 1.01–1.31; *P* = 0.04), platelet count (aHR, 0.97; 95% CI, 0.93–1.01; *P* = 0.20), and DAP dose (aHR, 0.70; 95% CI, 0.52–0.95; *P* = 0.02).

### Influence of DAP dose on the effect of DAP + BLA

We also examined the effect of DAP dose for patients given DAP + BLA using a multivariable Cox proportional hazard model analysis. To make the hazard model more clinically relevant, we separated patients receiving high-dose DAP (≥9 mg/kg) and low-dose DAP (<9 mg/kg). The results show that patients given high-dose DAP + BLA had significantly better survival than those receiving low-dose DAP alone (aHR, 5.16; *P* = 0.02), low-dose DAP + BLA (aHR, 5.39; *P* = 0.006), and high-dose DAP alone (aHR, 19.01; *P* = 0.002) (Model 2 in Table 3, Fig. 2B). Among the 70 survivors, high-dose DAP + BLA group received 13 (12–13) days of daptomycin therapy, low-dose DAP alone 13 (11–15) days, low-dose DAP + BLA 13 (11–19) days, and high-dose DAP alone 5 (4–6) days (*P* = 0.17). There was no significant association between the cardiac echo examination and the 4 treatment groups (*P* = 0.27). Two patients with proven endocarditis were included in the study. One patient received high-dose DAP + BLA, and the other one received low-dose DAP + BLA. Both of them survived. There was no significant association between the diagnosis of endocarditis and the 4 treatment groups (*P* = 0.68) and the mortality (*P* = 0.52).

### Sensitivity analysis

We defined deceased patients and the 4 other patients whose antibiotics were switched to linezolid due to persistent BSI under DAP treatment as DAP treatment failure (Fig. 1), and used them as the cohort for sensitivity analysis. For patients with DAP MICs of 2 mg/L or less, multivariable analysis indicated the DAP + BLA group had a significantly lower treatment failure rate (aHR, 0.23; 95% CI, 0.06–0.91; *P* = 0.04). Patients receiving high-dose DAP + BLA had a significantly lower rate of treatment failure than those receiving low-dose DAP alone (*P* = 0.04), low-dose DAP + BLA *(P* = 0.02), and high-dose DAP alone (*P* = 0.004).

We also used time-dependent multivariable Cox proportional hazard model for sensitivity analysis. The use of DAP + BLA was not significantly associated with mortality when the influence of DAP MIC and DAP dose were not considered in the adjusted model (aHR, 0.71; 95% CI, 0.38–1.34; *P* = 0.29; Model 3 in Table 4). However, use of DAP + BLA significantly decreased mortality when the DAP MIC was 2 mg/L or less (aHR, 0.23; 95% CI, 0.06–0.81; *P* = 0.02), but not when the MIC was 4 mg/L or more (aHR, 1.13; 95% CI, 0.51–2.50; *P* = 0.76). Patients receiving high-dose DAP + BAL had significantly better survival than those receiving low-dose DAP alone (aHR, 13.79; *P* = 0.01), low-dose DAP + BLA (aHR, 13.05; *P* = 0.01), and high-dose DAP alone (aHR, 17.64; *P* = 0.01) (Model 4 in Table 4, Fig. 2C).

**Table 4:** Time-dependent Cox Proportional Hazards Model of Factors Associated with Mortality.

	Adjusted Model 3^a^			Adjusted Model 4^c,d^	
	Hazard ratio (95% CI)	*P*		Hazard ratio (95% CI)	*P*
Steroid use	2.85 (1.42–5.71)	0.003	Steroid use	3.51 (1.75–7.04)	<0.001
Pitt bacteremia score	1.17 (1.05–1.31)	0.003	Pitt bacteremia score	1.15 (1.04–1.29)	0.008
Platelet count (×10^4^/μL)	0.96 (0.92–0.99)	0.03	Platelet count (×10^4^/μL)	0.96 (0.92–1.00)	0.03
			Treatment regimens		
DAP dose (mg/kg)	0.75 (0.59–0.94)	0.01	DAP dose ≥9 mg/kg with BLA	Reference	
DAP + BLA	0.71 (0.38–1.34)	0.29^b^	DAP dose <9 mg/kg without BLA	13.79 (1.81–104.80)	0.01
			DAP dose <9 mg/kg with BLA	13.05 (1.73–98.15)	0.01
			DAP dose ≥9 mg/kg without BLA	17.64 (1.92–161.90)	0.01

Abbreviations: BLA, beta-lactam antibiotic; CI, confidence interval; DAP, daptomycin.

^a^Test of proportional-hazards assumption: *P* = 0.98.

^b^DAP + BLA was forced as an independent variable in the final adjusted model 3.

^c^Test of proportional-hazards assumption: *P* = 0.97.

^d^Interactions between daptomycin dose and beta-lactam combinations were considered in the final adjusted model 4.

## Discussion

After careful evaluation of the possible influence of DAP dose on the effect of a DAP + BLA regimen, we found that patients receiving high-dose DAP + BLA independently had a better survival than those receiving low-dose DAP alone, low-dose DAP + BLA, and high-dose DAP alone. These findings are supported by our sensitivity analysis, which used time-dependent Cox proportional hazard model analysis.

When DAP is combined with a BLA, even low-dose DAP had comparable or even more rapid bacterial killing than high-dose DAP alone^8,9^. However, some of the previous clinical reports of DAP and BLA combinations are contradictory^14,15,29^. Only few clinical studies compared the effect of DAP + BLA with DAP alone in treating VRE-BSI^5,18,19^. These studies showed that a DAP + BLA regimen was not associated with less microbiology failure, not associated with better BSI clearance^18^, and not associated with lower mortality^5,19^. There are several problems with these studies related to the MICs of the isolates and the dose of DAP. The synergistic interactions of DAP and BLA depend on the DAP MICs and the DAP dose^9,10^. In our cohort, we showed that the high-dose DAP + BLA was associated with better survival than the other regimens (even high-dose DAP alone). The optimal dose of DAP remains to be defined, although previous studies suggested more than 9 or 10 mg/kg may be optimal^5,7^. Whether DAP + BLA provides additional survival benefit with use of an even higher dose of DAP (*e.g*. 12 or more mg/kg) remains unknown^9^.

By testing the effect of DAP + BLA with stratified by DAP MIC, we found that the beneficial effect might be most evident when DAP MIC was 2 mg/L or less. This result should be viewed with caution due to our small sample size, and the result was obtained from a subgroup analysis. Though, other *in vitro* studies showed that ampicillin or ceftriaxone provided no benefit to DAP treatment when there was a high DAP MIC, but the high DAP MIC cut-offs were 32 mg/L and 10 mg/L respectively^9,10^. Another limitation of our study is that not all BLA MICs were available. Whether MIC of the combined BLA also influent the survival benefit of DAP + BLA remained unknown.

One dose of BLA might not be comfortably considered to have meaningful effect. However, several recent studies have used the definition of at least one dose of BLA as the definition of combination therapy in investigating this topic^5,19^. In addition, using the definition of combination duration cut-offs such as at least 3 days of BLA combinations would result in a more bias conclusion to favor the combination therapy, since by such definitions the combinations group would have to be at least alive for 3 days and thus leading to more immortal time bias. Only three patients died on the same day of the DAP + BLA combinations. Most of the DAP + BLA patients (76/87) had the BLA combination since the day that daptomycin was started, and the median duration of the BLA combinations of these 76 patients was 9 days (IQR: 6–13). Furthermore, even we used the definition that only patients who received at least 3 days of BLA combination throughout the daptomycin treatment course for the combination group, the results remained similar in that patients given high-dose DAP + BLA had significantly better survival than those receiving low-dose DAP alone (aHR, 5.82; *P* = 0.01), low-dose DAP + BLA (aHR, 3.99; *P* = 0.03), and high-dose DAP alone (aHR, 26.61; *P* = 0.001).

A major problem in evaluating the effect of the DAP + BLA regimen in a cohort study is the “immortal time” bias, *i.e*. patients who live longer are more likely to receive BLA, and this might bias the results in favor of the DAP + BLA regimen^28^. The finding that among the DAP + BLA group, the non-survivors received shorter BLA combinations compared to the survivors might be due to a dose-dependent response of the DAP + BLA combinations or an immortal time bias. One of the strengths of this study is that we examined the treatment regimen (DAP *vs*. DAP + BLA) as a time-dependent variable in the sensitivity analysis to circumvent possible immortal time bias^28^. Nonetheless, there might still be uncontrolled cofounding by indications, meaning that patients who are sicker might be more likely to receive DAP + BLA. However, this would lead to a more conservative estimate of the effect of DAP + BLA, meaning that our results under-estimated the benefit of the DAP + BLA regimen. In the present study, the major results of the main analysis and the sensitivity analysis are consistent. Our study also carefully examined the influence of important treatment-effect modifying variables, namely, the DAP doses and DAP MICs. The current findings provide the necessary assurance to conduct a randomized, controlled, clinical trial examining the effect of DAP + BLA as a treatment for VRE-BSI.

This study has several limitations that were due to its observational design. Although we used multivariable analysis to adjust for possible confounders of mortality, residual unmeasured confounding variables might still present. Due to the nature of observational study design, the collection of follow-up bacterial cultures depended on the primary care physicians. Therefore, we could not evaluate differences in the microbiological responses, such as bacterial clearance or resistance prevention. Though, we identified that the only significant difference between the DAP and DAP + BLA groups was that patients in the DAP group had a higher percentage of recent chemotherapy. Recent chemotherapy didn’t independently predict mortality. Another significant limitation of our study is that it is unclear why BLA were administered to certain patients or why the type and duration of BLA therapy was chosen as this was determined by primary care physicians. Only few patients received solid organ transplants or hematopoietic stem cell transplants, so the effect of DAP + BLA in these patients warrants further validation. Because of our relatively small sample size, we could not evaluate the interaction of DAP + BLA, DAP dose, and DAP MIC all together. In addition, patients received many different BLAs in combination with DAP, so we could not identify the single most effective BLA, and the effect of the dose of each BLA.

In conclusion, use of a higher dose of DAP, and use of a DAP + BLA regimen were associated with better survival of patients with VRE-BSI. In addition, the DAP MIC and DAP dose influenced the effectiveness of the DAP + BLA regimen. The DAP + BLA regimen might improve survival compared to DAP alone when the DAP MIC was 2 mg/L or less, even after adjustment for DAP dose. VRE-BSI patients who received a DAP dose of 9 mg/kg or more and also received a BLA had better survival than all other groups. Due to the nature of observational study design, the results should be interpreted cautiously. Adequately sized prospective controlled clinical trials, employing the most effective DAP-BLA combinations based on *in vitro* studies and dosage, are needed to validate the efficacy of DAP + BLA regimens for the treatment of VRE-BSI.

## Electronic supplementary material

Supplementary Table S1
