# Supplementary material for: A retrospective clinical comparison of daptomycin vs daptomycin and a beta-lactam antibiotic for treating vancomycin-resistant Enterococcus faecium bloodstream infections
Source: Sci Rep. 2018 Jan 26;8:1632. doi: 10.1038/s41598-018-19986-8 (PMC5786011; doi:10.1038/s41598-018-19986-8)
Supplement: Supplementary file 1 — Supplementary Table S1 [file 41598_2018_19986_MOESM1_ESM.pdf]

Online Supplementary Information

A retrospective clinical comparison of daptomycin vs daptomycin and a beta-lactam antibiotic for treating vancomycin-resistant *Enterococcus faecium* bloodstream infections

Yu-Chung Chuang [1,2], Pao-Yu Chen [3], Chi-Ying Lin [4], Yee-Chun Chen[2], Jann-Tay Wang*[2a], Shan-Chwen Chang [2a]

[1]Graduate Institute of Clinical Medicine, College of Medicine, National Taiwan University, Taipei, Taiwan; [2]Department of Internal Medicine, National Taiwan University Hospital, Taipei, Taiwan; [3]Department of Traumatology, National Taiwan University Hospital, Taipei, Taiwan; [4]Department of Internal Medicine, National Taiwan University Hospital Yun-Lin Branch, Yun-Lin, Taiwan.

[a] J.-T. Wang and S.-C. Chang are co-corresponding authors.

***Correspondence:**
Dr. Jann-Tay Wang, Department of Internal Medicine, National Taiwan University Hospital, Taipei, Taiwan, 7 Chung-Shan South Road, Taipei, Taiwan 100
Telephone: 886-2-23123456 ext. 65054; Fax: 886-2-23971412
E-mail: 14bcr@yahoo.com.tw

Supplementary Table S1. Beta-lactam combination therapy for each of the 87 patients.

Patient	days of daptomycin treatment	Beta-lactam antibiotics combination [a,b]
ID15	8	TZP 3000mg q6h, D3-D5; IPM 1000mg q6h, D5-D8
ID23	3	CAZ 2000mg q8h, D1-D2
ID29	10	FEP 2000mg q8h, D1-D10
ID35	2	MEM 1000mg q8h, D1-D2
ID36	13	CAZ 2000mg q8h, D1-D13
ID42	2	IPM 1000mg q6h, D1-D2
ID46	14	FEP 2000mg q8h, D1-D3; CAZ 2000mg q8h, D4-D14
ID64	22	FEP 2000mg q8h, D1-D9; IPM 1000mg q6h, D9-D9
ID65	15	CAZ 2000mg q8h, D1-D9; MEM 1000mg q8h, D9-D15
ID89	4	IPM 1000mg q6h, D4-D4
ID90	4	TZP 3000mg q6h, D1-D2; IPM 1000mg q6h, D2-D4
ID94	4	CAZ 2000mg q8h, D3-D3; IPM 1000mg q6h, D4-D4
ID96	3	MEM 1000mg q8h, D1-D3
ID102	62	IPM 1000mg q6h, D7-D21; FEP 2000mg q8h, D30-D31
ID104	9	TZP 3000mg q6h, D1-D9
ID105	7	IPM 1000mg q6h, D1-D2
ID106	5	IPM 1000mg q6h, D1-D5
ID107	8	MEM 1000mg q8h, D1-D7
ID109	20	CAZ 2000mg q8h, D1-D20
ID112	7	CAZ 2000mg q8h, D1-D7
ID114	12	CMZ 2000mg q8h, D1-D9
ID117	8	FEP 2000mg q8h, D1-D6
ID119	1	MEM 1000mg q8h, D1-D1
ID120	5	CAZ 2000mg q8h, D1-D3; MEM 1000mg q8h, D3-D5
ID128	14	MEM 1000mg q8h, D1-D5; MEM 1000mg q8h, D13-D14
ID144	32	IPM 1000mg q6h, D1-D9; IPM 1000mg q6h, D17-D30
ID145	15	FEP 2000mg q8h, D1-D15
ID146	23	CAZ 2000mg q8h, D1-D14
ID147	10	IPM 1000mg q6h, D1-D6
ID150	15	MEM 1000mg q8h, D1-D4; FEP 2000mg q8h, D11-D15
ID153	12	MEM 1000mg q8h, D1-D6
ID154	12	IPM 1000mg q6h, D1-D9

ID155	14	MEM 1000mg q8h, D1-D6; CAZ 2000mg q8h, D6-D14
ID157	15	TZP 3000mg q6h, D1-D7; CAZ 2000mg q8h, D9-D15
ID158	10	TZP 3000mg q6h, D1-D4
ID159	12	CAZ 2000mg q8h, D1-D9; FEP 2000mg q8h, D9-D10; MEM 1000mg q8h, D10-D12
ID160	5	ETP 1000mg qd, D1-D3
ID164	9	CAZ 2000mg q8h, D1-D5; CAZ 2000mg q8h, D9-D9
ID167	1	MEM 1000mg q8h, D1-D1
ID169	40	IPM 1000mg q6h, D1-D5; CAZ 2000mg q8h, D5-D10; CAZ 2000mg q8h, D27-D40
ID181	9	TZP 3000mg q6h, D1-D5; AMC 1000mg q6h, D5-D6; FEP 2000mg q8h, D5-D9
ID184	14	MEM 1000mg q8h, D1-D12
ID183	3	MEM 1000mg q8h, D1-D3
ID185	13	CRO 1000mg q12h, D1-D13
ID190	13	MEM 1000mg q8h, D1-D8
ID193	14	ETP 1000mg qd, D1-D2; IPM 1000mg q6h, D2-D14
ID192	20	FEP 2000mg q8h, D8-D12
ID194	13	MEM 1000mg q8h, D1-D10
ID201	4	MEM 1000mg q8h, D1-D4
ID202	13	IPM 1000mg q6h, D1-D8; CMZ 2000mg q8h, D8-D13
ID205	2	IPM 1000mg q6h, D1-D2
ID204	9	MEM 1000mg q8h, D1-D8
ID206	17	MEM 1000mg q8h, D1-D17
ID208	15	MEM 1000mg q8h, D1-D13
ID209	27	MEM 1000mg q8h, D1-D12; MEM 1000mg q8h, D20-D27
ID212	22	CAZ 2000mg q8h, D9-D9; MEM 1000mg q8h, D9-D22
ID217	4	MEM 1000mg q8h, D1-D4
ID222	39	MEM 1000mg q8h, D30-D39
ID226	13	MEM 1000mg q8h, D1-D13
ID229	15	CAZ 2000mg q8h, D1-D11; CAZ 2000mg q8h, D13-D15
ID234	16	CAZ 2000mg q8h, D1-D6; IPM 1000mg q6h, D6-D7; MEM 1000mg q8h, D7-D13
ID239	8	MEM 1000mg q8h, D1-D6
ID240	14	CAZ 2000mg q8h, D1-D14
ID244	13	CAZ 2000mg q8h, D1-D3; TZP 3000mg q6h, D3-D9
ID250	28	IPM 1000mg q6h, D1-D13; MEM 1000mg q8h, D16-D28
ID249	11	CAZ 2000mg q8h, D1-D11

ID252	22	FEP 2000mg q8h, D1-D3; MEM 1000mg q8h, D3-D22
ID256	14	MEM 1000mg q8h, D1-D4; CAZ 2000mg q8h, D13-D14
ID257	8	IPM 1000mg q6h, D2-D8
ID260	9	MEM 1000mg q8h, D1-D9
ID262	13	TZP 3000mg q6h, D1-D2; FEP 2000mg q8h, D2-D9
ID263	13	IPM 1000mg q6h, D1-D10
ID265	14	FEP 2000mg q8h, D1-D9
ID272	10	MEM 1000mg q8h, D4-D10
ID276	11	FEP 2000mg q8h, D1-D6
ID278	12	TZP 3000mg q6h, D1-D2
ID280	13	FEP 2000mg q8h, D6-D7; ETP 1000mg qd, D7-D13
ID285	14	MEM 1000mg q8h, D1-D14
ID286	20	ETP 1000mg qd, D1-D17; MEM 1000mg q8h, D17-D20
ID292	2	TZP 3000mg q6h, D1-D2
ID293	5	CSL 2000mg q12h, D4-D5
ID295	10	MEM 1000mg q8h, D1-D5; FEP 2000mg q8h, D5-D10
ID294	15	CAZ 2000mg q8h, D1-D7
ID298	8	MEM 1000mg q8h, D1-D8
ID304	13	MEM 1000mg q8h, D1-D13
ID305	22	ETP 1000mg qd, D1-D16
ID309	11	FEP 2000mg q8h, D1-D9; CAZ 2000mg q8h, D11-D11

Abbreviations:

AMC, amoxicillin-clavulanic acid; CAZ, ceftazidime; CMZ, cefmetazole; CRO, ceftriaxone; CSL, cefoperazone-sulbactam; ETP, Ertapenem, FEP, cefepime; IPM, imipenem; MEM, meropenem ; TZP, piperacillin-tazobactam

[a] Day 1 (D1) was the day on which the daptomycin starts

[b] there are dose adjustments for renal impairment
